# CMCL-DDI: Pharmacophore-aware cross-view contrastive learning for drug-drug interaction prediction

**DOI:** 10.1371/journal.pone.0341952

**Published:** 2026-02-23

**Authors:** Yehong Han, Lin Du

**Affiliations:** School of Information Science and Engineering, Qilu Normal University, Jinan, Shandong, China; Karlstad University: Karlstads Universitet, SWEDEN

## Abstract

Accurate prediction of potential drug-drug interactions (DDIs) is vital for ensuring medication safety and efficacy. Existing graph-based methods typically focus on molecular structures but often overlook the complementary semantic information embedded in SMILES (Simplified Molecular Input Line Entry System) representations. To address this gap, we propose CMCL-DDI, a Cross-view Mutual Contrastive Learning framework that jointly leverages pharmacophore-aware molecular graphs and SMILES sequences. Specifically, we encode pharmacophore-based subgraphs to capture functional molecular features and aggregate them into expressive graph-level embeddings. In parallel, SMILES sequences are encoded to preserve sequential drug characteristics. A contrastive learning strategy aligns both views in a shared latent space, facilitating mutual representation enhancement. Furthermore, we design a cross-attention fusion module to integrate heterogeneous features, enabling robust and interpretable DDI prediction. Extensive experiments on benchmark datasets demonstrate that CMCL-DDI consistently outperforms state-of-the-art models, highlighting the effectiveness of cross-view representation learning for DDI prediction. The source codes are available at https://github.com/95LY/CMCL-DDI.

## Introduction

Drug-drug interactions (DDIs) describe the potential effects that may arise when two or more drugs are administered together. Investigating DDIs is a vital component of both drug development and clinical practice, aiming to uncover and assess possible interactions to ensure safe and effective pharmacotherapy [1]. In managing complex diseases, the concurrent use of multiple medications is often required. However, such polypharmacy can significantly alter pharmacological responses, potentially affecting therapeutic efficacy. While multi-drug regimens may enhance treatment outcomes by leveraging the synergistic effects of different drugs, they also pose increased risks of adverse reactions, which in severe cases can be life-threatening [2–4]. Therefore, in clinical practice, the ability to accurately predict DDIs is of considerable importance [5,6]. Effective DDI prediction helps minimize adverse drug events [7], thereby reducing patient hospitalizations, lowering healthcare expenditures, and preventing treatment failures. Moreover, it assists healthcare providers in identifying potentially hazardous drug combinations, enabling safer and more informed prescribing decisions. Artificial Intelligence (AI) and Deep Learning (DL) techniques have achieved significant advances in tackling complex problems in bioinformatics [8]. These approaches offer powerful tools for drug discovery and predicting interactions between existing drugs by enabling efficient analysis of complex biomedical data [8,9]. Traditional methods, limited by insufficient biochemical information, often struggle to model deep structural relationships and scale to large datasets. In response, a variety of deep learning-based computational frameworks have been developed, demonstrating strong performance in DDI prediction and attracting growing attention within the research community.

Graph Neural Networks (GNNs), such as GCN(Graph Convolutional Network) [10], GAT(Graph Attention Network) [11], and GIN(Graph Isomorphism Network) [12], have been extensively applied to DDI prediction tasks [13–17]. In molecular graph-based approaches, drugs are represented as graphs derived from their SMILES (Simplified Molecular Input Line Entry System) strings [18], typically converted via the RDKit toolkit [19]. In these graphs, atoms and chemical bonds are represented as nodes and edges, respectively [20–23]. For instance, Deac et al. [24] proposed a GNN framework that leverages molecular structural information for DDI prediction. Similarly, Wang et al. [25] combined pharmaceutical and genomic features with GCN and attention mechanisms to identify synergistic drug combinations. In another study, Zhang et al. [26] incorporated node centrality, spatial encoding, and edge descriptors, along with a lightweight attention module, to capture structural properties of drug molecules. Furthermore, drugs can be decomposed into bioactive substructures—such as specific functional groups or atom clusters—which play essential roles in DDI modeling. Several works have focused on learning substructure-level interactions to improve prediction accuracy [27–30]. For example, Nyamabo et al. [31] employed GAT to process molecular graphs of drug pairs and extract substructure representations within the receptive fields of each layer. Similarly, Yu et al. [32] constructed substructure-aware embeddings using predefined functional groups and designed a tensor neural network tailored for DDI prediction.

Recent advances have demonstrated the effectiveness of multi-view based DDI prediction, where drugs are represented using multiple modalities such as molecular graphs, SMILES strings, and 3D structures. Leveraging these diverse features simultaneously can significantly improve predictive performance. Liu et al. [33] introduced MFFGNN, which integrates topological information from molecular graphs and SMILES through feature extraction modules (MGFEM and SSFEM), followed by aggregation and fusion to enhance drug representations. Similarly, Song et al. [34] proposed AMDE, which encodes drug features in multiple dimensions. Their method uses two channels to process drug SMILES sequences, extracting two-dimensional atom map features and one-dimensional sequence features using Rdkit and FCS, respectively. These features are then sent to a 2D feature graph encoder and a 1D feature sequence encoder for further encoding. On the other hand, Chen et al. [35] proposed 3DGT-DDI, which combines 3D structural features with textual information using a 3D GNN and textual attention mechanism. SCIBERT is employed for extracting text features, while SchNet [36] captures 3D geometric data, enhancing prediction accuracy and model interpretability.

Another promising direction in DDI research is contrastive learning based DDI prediction, which has gained popularity for its ability to learn discriminative and informative representations. Wu et al. [37] introduced MIRACLE, a graph-centric contrastive learning framework that aligns intra- and inter-view representations within structural modalities. However, it does not consider cross-modal alignment (e.g., between SMILES and molecular graphs), limiting its capacity to explore complementary semantic information across different modalities. To align and integrate features from different views, MIRACLE utilizes a Jensen–Shannon-based mutual information estimator [38], which allows the model to generate more informative and discriminative embeddings by focusing on key substructures and filtering out irrelevant noise. Similarly, DSN-DDI [39] employs both intra-view (single-drug graph) and inter-view (bipartite drug-drug graph) representations, with feature propagation performed within individual drugs and across drugs to jointly model their contextual dependencies. These methods highlight the advantages of multi-view integration and contrastive learning in improving DDI prediction.

Unlike the aforementioned studies [33–37], our proposed CMCL-DDI framework introduces a cross-modal mutual contrastive learning strategy that explicitly models the interaction and consistency between molecular graphs and SMILES representations, rather than processing them independently. While existing approaches typically fuse features from multiple modalities through concatenation or shallow attention mechanisms, CMCL-DDI performs mutual contrastive alignment between the two modalities to achieve cross-view enhancement. Moreover, our graph encoder incorporates pharmacophore-level structural priors, enabling the model to capture chemically meaningful substructures that are often overlooked in prior works. This design not only strengthens semantic interaction between modalities but also improves interpretability, robustness, and predictive reliability in DDI tasks.

Despite advancements in DDI prediction, several challenges remain: (1) Existing methods often treat molecular graphs and SMILES sequences independently, lacking mechanisms for mutual enhancement, which limits the depth of cross-view representation learning. (2) Most graph-based encoders overlook pharmacophore-level information—key substructures linked to drug activity—thereby missing crucial chemical semantics essential for accurate and interpretable predictions. (3) Current fusion strategies typically use simple concatenation or shallow attention, failing to capture complex inter-view dependencies, which hinders model robustness and generalization.

To address the aforementioned challenges, we propose CMCL-DDI, a novel framework that integrates molecular structural and semantic information through cross-view contrastive learning. This approach enables the model to fully exploit complementary features across views, enhancing the robustness of DDI prediction. Specifically, CMCL-DDI captures graph-level representations from pharmacophore-aware molecular graphs and semantic-level features from SMILES sequences. During the DDI prediction stage, a cross-attention mechanism is employed to effectively fuse the structural and semantic representations, allowing for more accurate and interpretable predictions.

The main contributions of this study are summarized as follows:

1. We propose a cross-view contrastive learning framework that enables mutual enhancement between drug molecular graphs and SMILES strings, improving representation quality.
2. We encode pharmacophore-based subgraphs and aggregate them into graph-level embeddings, enhancing the model’s ability to capture functional drug features for more accurate and interpretable DDI prediction.
3. We introduce a cross-attention fusion module to integrate complementary features from molecular graphs and SMILES, enabling more robust and accurate DDI prediction.
4. Our method achieves state-of-the-art results on standard DDI prediction datasets, outperforming existing models and demonstrating superior predictive power.

## Methods

**Fig 1:**
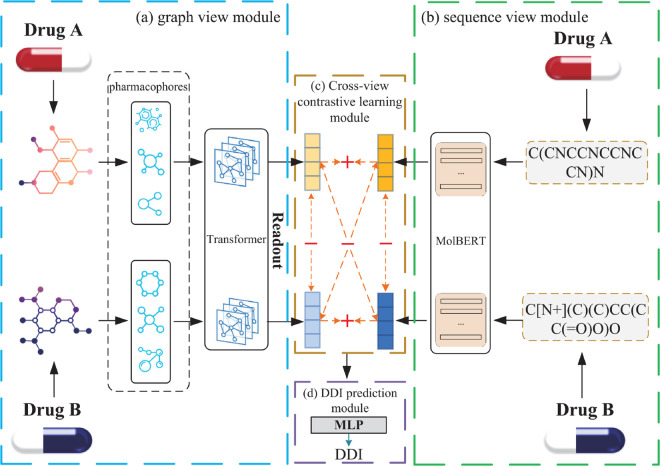
Overview of the CMCL-DDI framework. It comprises three main components: (a) graph view module, which encodes pharmacophore-level structural features using a Transformer-based encoder and readout function. Each pharmacophore box corresponds to the subgraphs decomposed from a single molecule; (b) sequence view module, which extracts semantic representations from SMILES strings using MOLBERT; and (c) cross-view contrastive learning module, which aligns the representations from both views during training. (d) DDI prediction module, which fuses the learned representations via a cross-attention mechanism and predicts interaction probability using a multi-layer perceptron.

As illustrated in Fig 1, we propose CMCL-DDI, a cross-modal contrastive learning framework for DDI prediction, which consists of three key components: (a) graph view module, (b) sequence view module, and (c) cross-view contrastive learning. In the graph view module, drug molecules are decomposed into pharmacophores and encoded using a Transformer-based encoder, followed by a readout operation to generate structural drug representations. In the sequence view module, SMILES strings are encoded via MOLBERT to extract semantic representations.

During training, CMCL-DDI employs a cross-view contrastive learning objective to align and enhance the consistency between the two views. Specifically, each drug is represented in both the molecular graph view and the SMILES view. For each training instance, representations of the same drug across two views are treated as a positive pair, while representations of different drugs form negative pairs. The model is optimized to maximize the agreement of positive pairs and minimize that of negative pairs using a contrastive loss. This process encourages the model to align semantically consistent representations across modalities while distinguishing heterogeneous drugs, thereby enabling mutual information exchange and improving the discriminative power of learned embeddings.

After training, the learned representations from both views are fused through a cross-attention mechanism to predict potential drug-drug interactions, enabling the model to effectively leverage complementary structural and sequential information for accurate DDI inference.

### Graph view module

The molecular structure of a drug plays a crucial role in determining its pharmacological behavior and interaction potential. To effectively leverage structural information for DDI prediction, we first represent each drug molecule as a graph, where atoms serve as nodes and chemical bonds as edges. Rather than utilizing the full molecular graph directly, we decompose the molecule into pharmacophore substructures, which capture essential substructures responsible for efficacy. These pharmacophores are fed into a Transformer encoder, and the pharmacophore features obtained are then processed through a readout function to obtain drug molecular features.

The molecular graph is denoted G={V,E}, *V* refers to the set of nodes, and *E* denotes the set of edges. We use the BRICS [40] to decompose molecules into several fragments with pharmacophore. Each molecule is represented as a graph G={V,E} and is decomposed into multiple pharmacophore subgraphs, rather than multiple molecules per box. The set of pharmacophores is represented as $G=\{(V^{1},E^{1}),\;(V^{2},E^{2}),\;(V^{3},E^{3}),\;\ldots ,\;(V^{N},E^{N})\}$, where *N* denotes the total number of pharmacophores. Taking the first pharmacophore (*V*^1^,*E*^1^) as an example, $v_{\left(i\right)}^{1}\in V^{1}$ represents the *i*-th atom ($1\leq i\leq |V^{1}|$) and $e_{\left(j\right)}^{1}\in E^{1}$ denotes the *j*-th bond ($1\leq j\leq |E^{1}|$). The node feature matrix of the first pharmacophore is denoted as $X_{V}^{1}\in {\mathbb{R}}^{|V^{1}|\times D_{V}}$, where each row is the feature vector of a node (i.e., an atom) encoding its type (H, C, O, N). The edge feature matrix is denoted as $X_{E}^{1}\in {\mathbb{R}}^{|E^{1}|\times D_{E}}$, where each row is the feature vector of an edge (i.e., a chemical bond) encoding its type (single, double, triple, etc.). Here, $D_{V}$ and *D*_*E*_ indicate the dimensions of the node and edge feature vectors, respectively.

We first project the initial node and edge features into a common latent space, yielding the updated features $X_{V^{\prime }}^{1}\in {\mathbb{R}}^{|V^{1}|\times d}$ and $X_{E^{\prime }}^{1}\in {\mathbb{R}}^{|E^{1}|\times d}$, where *d* denotes the unified feature dimension. The initial pharmacophore representation is then obtained by concatenating these features -wise:

$$X_{1}^{\prime }=[\;X_{V^{\prime }}^{1}\;\|\;X_{E^{\prime }}^{1}\;]\in {\mathbb{R}}^{(|V^{1}|+|E^{1}|)\times d}$$ (1)

where [·‖·] denotes -wise concatenation. This fused representation $X_{1}^{\prime }$ serves as the input to the subsequent graph encoder, capturing both node-level and edge-level information for each pharmacophore.

**Fig 2:**
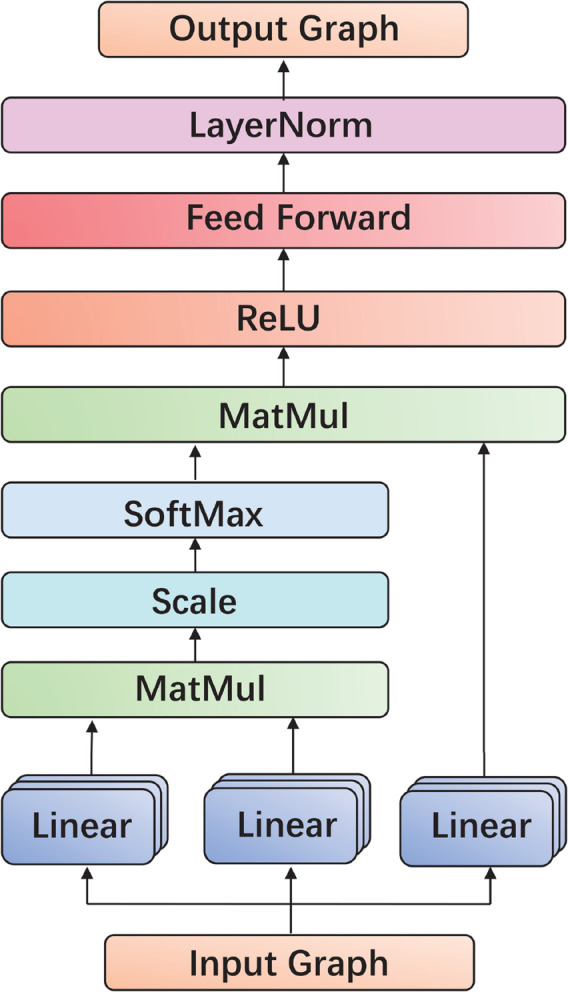
The architecture of transformer.

Next, we employ a Transformer to encode the initial features of the first functional group, aiming to obtain higher-level feature representations. The Transformer architecture is shown in Fig 2.

$$Q_{i}=X_{1}^{\prime }W_{Q}^{\;2pt(i)}$$ (2)

$$K_{i}=X_{1}^{\prime }W_{K}^{\;2pt(i)}$$ (3)

$$V_{i}=X_{1}^{\prime }W_{V}^{\;2pt(i)}$$ (4)

$$Attention_{i}(Q_{i},K_{i},V_{i})=softmax(\frac{Q_{i}K_{i}^{T}}{\sqrt{d_{k}}})V_{i}$$ (5)

$$X_{1}^{\;{1.5pt}^{\prime \prime }}=MultiHead(Q,K,V)=Concat(Attention_{1},...,Attention_{h})W^{\;1.5ptO}$$ (6)

where $W_{Q}^{\;2pt(i)}$, $W_{K}^{\;2pt(i)}$, $W_{V}^{\;2pt(i)}$, *W^O^* represent learnable matrices.

By repeating the same process, we obtain the features of all pharmacophores in the molecule: $X_{2}^{\;{1.5pt}^{\prime \prime }}$, $X_{3}^{\;{1.5pt}^{\prime \prime }}$,..., $X_{N}^{\;{1.5pt}^{\prime \prime }}$.

Next, we perform a readout operation on the features of all pharmacophores contained in the drug molecule to obtain its molecular feature. The detailed process is described as follows.

$$z_{mol}=Readout(X_{1}^{\;{1.5pt}^{\prime \prime }},X_{2}^{\;{1.5pt}^{\prime \prime }},X_{3}^{\;{1.5pt}^{\prime \prime }},...,X_{N}^{\;{1.5pt}^{\prime \prime }})$$ (7)

### Sequence view module

To extract representations of drug molecules from sequence view, we employ a BERT-based encoder to process their SMILES strings. Given a SMILES sequence $S=(s_{1},s_{2},...,s_{n})$, where *s*_*i*_ denotes the *i*-th token in the sequence, the BERT encoder maps *S* into a sequence of contextualized embeddings:

$$H=MolBERT(S)=({\text{h}}_{1},h_{2},...,h_{n})$$ (8)

where $h_{i}\in {\mathbb{R}}^{d}$ represents the hidden feature vector of the *i*-th token, and *d* denotes the embedding dimension.

For example, for the SMILES sequence CCO, the tokenized sequence is S=(C,C,O), and MolBERT produces the embeddings:

$$H=[\begin{array}{c} h_{1}\\ h_{2}\\ h_{3} \end{array}]\in {\mathbb{R}}^{3\times d}$$ (9)

where each *h*_*i*_ is a *d*-dimensional vector that encodes the corresponding atom together with its sequential context.

Internally, the MolBERT encoder works as follows: 1) each token is first mapped to an embedding vector and combined with a positional encoding; 2) the sequence of embeddings is then processed by multiple self-attention layers of the Transformer, which allows each token to attend to all other tokens in the sequence; 3) the output is a sequence of contextualized embeddings *H*, which can be used as input to downstream modules.

To obtain a fixed-dimensional molecular representation, we apply a readout function over the sequence of token embeddings. Specifically, we adopt mean pooling across all token representations:

$$h_{mol}=\frac{1}{n}\sum_{i=1}^{n}h_{i}$$ (10)

where $h_{mol}\in {\mathbb{R}}^{d}$ denotes the final feature vector representing the entire drug molecule.

### Cross-view contrastive learning module

In this study, we employ a cross-view contrastive learning strategy to train feature representations for graph and sequence views. Specifically, our goal is to maximize the similarity between the features of the same drug across different views (graph and sequence views), while minimizing the similarity between different drugs within these views, thereby optimizing the multimodal representation of the drugs.

For each drug molecule, we first extract the graph view feature $z_{mol}^{A}$ and sequence view feature $h_{mol}^{A}$. The graph view feature $z_{mol}^{A}$ are generated by a graph neural network encoder, while the sequence view feature $h_{mol}^{A}$ is generated by a MolBERT encoder. Similarly, for drug B, we extract the graph view feature $z_{mol}^{B}$ and sequence view feature $h_{mol}^{B}$.

The specific cross-view contrastive loss is as follows.

$$L_{cross}=-\log\frac{\exp(sim(z_{mol}^{A},{\text{h}}_{mol}^{A})/\tau )+\exp(sim(z_{mol}^{B},{\text{h}}_{mol}^{B})/\tau )}{\exp(sim(z_{mol}^{A},z_{mol}^{B})/\tau )+\exp(sim(z_{mol}^{A},{\text{h}}_{mol}^{B})/\tau )+\exp(sim({\text{h}}_{mol}^{A},z_{mol}^{B})/\tau )+\exp(sim(h_{mol}^{A},{\text{h}}_{mol}^{B})/\tau )}$$ (11)

where sim(·,·) denotes the cosine similarity and *τ* is a temperature parameter. The numerator contains the similarities of positive pairs, i.e., embeddings of the same molecule across augmentations or views, which the loss encourages to be high. The denominator additionally includes all negative pairs, i.e., combinations of embeddings that should not match, which the loss encourages to have low similarity. Intuitively, this loss pulls together embeddings of the same molecule across different views while pushing apart embeddings from different molecules or augmentations. This cross-view alignment enables the model to learn consistent molecular representations that integrate heterogeneous information from multiple modalities, improving downstream prediction performance.

### DDI prediction module

For each drug, we obtain a multimodal feature representation by concatenating its graph-level and sequence-level embeddings. Specifically, for drug A and drug B, the representations are calculated using the cross-attention module as follows:

$$d^{A}=CrossAtt(z_{mol}^{A},h_{mol}^{A})$$ (12)

$$d^{B}=CrossAtt(z_{mol}^{B},h_{mol}^{B})$$ (13)

The cross-attention fusion module explicitly models dependencies between molecular structure and sequence representations, facilitating the integration of heterogeneous information. Inspired by cross-attention fusion mechanisms in multimodal learning [41–43], this design enables mutual information exchange between structural and sequential embeddings. Compared to traditional fusion methods such as simple concatenation or averaging, our cross-attention-based integration captures fine-grained dependencies between molecular structure and sequence features, allowing CMCL-DDI to better identify potential interaction mechanisms.

The two drug representations are concatenated -wise and fed into a multi-layer perceptron (MLP) with a sigmoid activation to estimate the interaction probability.

$$\hat{y}=\sigma (MLP(Concat({\text{d}}^{A},{\text{d}}^{B})))$$ (14)

where σ(·) denotes the sigmoid function and MLP represents a feedforward neural network.

## Experiments

### Datasets

We assessed the performance of CMCL-DDI on two real-world datasets: DrugBank and TWOSIDES. DrugBank integrates bioinformatics, chemoinformatics, and other resources, providing comprehensive drug-related information [44]. It encompasses 86 distinct types of interactions, detailing how drugs influence the metabolism of others, and includes 1706 drugs with 191,808 DDI triplets. Each drug was represented by its SMILES string and transformed into a molecular graph using RDKit. For data splitting, we followed the warm-start and cold-start settings. The TWOSIDES dataset [45] consists of 645 drugs, 963 interaction categories, and 4,576,287 DDI triplets, curated through filtering and preprocessing of the original TWOSIDES data. Unlike DrugBank, the interactions in TWOSIDES are described at the phenotypic level.

### Experimental settings

To rigorously evaluate the performance of the DDI prediction model, we employ a 5-fold cross-validation strategy. The DDI prediction task is framed as a binary classification problem, where each instance comprises a pair of drugs annotated as either interacting or non-interacting. In the training phase, positive instances are assigned a label of “1,” while negative instances are labeled as “0.” Model training is conducted in accordance with the hyperparameter configurations detailed in Table 1.

**Table 1:** Hyperparameter configurations of model experiments.

Parameters	Value
Epoch	300
Learning rate	1e-3
Batch size	256
Weight decay	4e-4
Loss function	CL
Drug embedding dimension	64

### Evaluation metrics

In this section, we utilize three primary evaluation metrics-AUROC, AUPRC, and F1 score-to assess the performance of CMCL-DDI. The confusion matrix presented in Table 2 serves as the foundation for computing these metrics.

**Table 2:** Confusion matrix for prediction results.

	Actual Positive (P)	Actual Negative (N)
**Predicted Positive (P)**	True Positive (TP)	False Negative (FN)
**Predicted Negative (N)**	False Positive (FP)	True Negative (TN)

(1) Recall reflects the proportion of true positive instances correctly identified by a classification model. This metric becomes particularly crucial when the cost associated with false negatives (missed positive cases) is significant, as it aims to minimize the occurrence of such errors.

$$Recall=\frac{TP}{TP+FN}$$ (15)

(2) Accuracy is the proportion of correctly classified instances, including both true positives and true negatives, relative to the total number of instances in the dataset. This metric is particularly informative when the dataset is balanced, with an approximately equal distribution of positive and negative cases.

$$Accuracy=\frac{TP+TN}{TP+FN+FP+TN}$$ (16)

(3) Precision quantifies the proportion of true positive instances among all instances predicted as positive by a classification model. This metric is particularly significant when the cost of false positives (incorrectly identified positive cases) is high, as it seeks to minimize such errors.

$$Precision=\frac{TP}{TP+FP}$$ (17)

(4) The ROC curve is constructed on a coordinate system defined by the false positive rate (FPR) and the true positive rate (TPR). The area under the curve, referred to as AUROC, serves as a key metric for evaluating the model’s performance. A higher AUROC value indicates superior classification performance. The definitions of TPR and FPR are provided below.

$$TPR=\frac{TP}{TP+FN}$$ (18)

$$FPR=\frac{FP}{FP+TN}$$ (19)

(5) The Precision-Recall Curve (PRC) is generated by plotting the recall rate against the precision rate on a coordinate plane. The area under the PRC curve (AUPRC) serves as a quantitative measure of the model’s performance and is commonly used to evaluate the effectiveness of the classifier.

(6) F1 score is a metric that takes into account both Precision and Recall simultaneously. Its definition can be expressed as follows.

$$F1=\frac{2\times Precision\times Recall}{Precision+Recall}$$ (20)

(7) Statistical significance is assessed using the Kruskal–Wallis H test and the Mann–Whitney U test. The Kruskal–Wallis test evaluates whether there are overall differences among models for a given metric. If significant, pairwise comparisons are performed using the Mann–Whitney U test, with p-values adjusted by the Holm–Bonferroni method.

### Baselines

We evaluated CMCL-DDI against the current state-of-the-art methods. The baselines include substructure-based algorithms and dual-view representation learning methods.

- MHCADDI [46]: employs a co-attention mechanism to fuse the combined information of drug pairs, thereby enhancing the representation learning for each individual drug.
- SSI-DDI [47]: utilizes a multi-layer Graph Attention Network (GAT) to capture substructures and estimates the interaction probabilities between these substructures to predict drug-drug interactions (DDIs).
- MR-GNN [48]: employs a graph neural network (GNN) built on a multi-resolution architecture, coupled with a dual-graph state long short-term memory (LSTM) network, to predict entity interactions.
- GMPNN-CS [49]: captures substructure information at multiple scales and models the interactions between these substructures for predicting drug-drug interactions (DDI).
- GAT-DDI [50]: utilizes a graph attention network (GAT) to predict drug-drug interactions (DDIs) by capturing complex dependencies within the drug graph structure.
- DGNN-DDI [51]: employs a graph neural network (GNN) augmented with a substructure attention mechanism for the prediction of drug-drug interactions (DDIs).

### Performance evaluation

**Fig 3:**
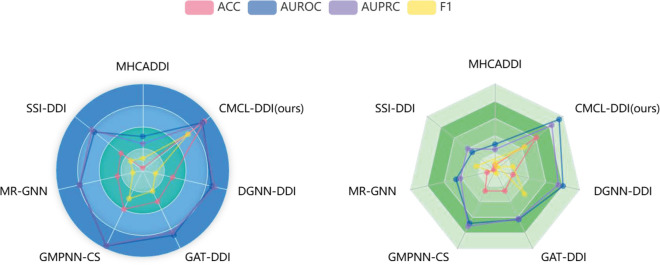
Performance comparison of different models under warm-start setting. The left figure displays results on the DrugBank dataset, and the right figure shows results on the Twosides dataset.

In the warm-start scenario, the training and testing sets share overlapping drugs. Each experiment is repeated 5 times, and the average performance across runs is reported. In each repetition, the dataset is randomly stratified into training, validation, and testing subsets while maintaining a balanced distribution of interaction types. To ensure fair comparisons among models, dataset splitting is performed prior to training, ensuring that all models are evaluated on identical data partitions. Table 3 presents the average performance of all models across the 5 runs. It is evident that CMCL-DDI consistently outperforms all baseline methods on both the DrugBank and TWOSIDES datasets across all evaluation metrics. Although previous state-of-the-art models achieve impressive accuracies of 96.33% and 86.96% on these datasets, respectively, CMCL-DDI further improves the results, reaching accuracies of 98.26% on DrugBank and 90.25% on TWOSIDES. In addition, CMCL-DDI attains outstanding AUPRC values of 99.25% on DrugBank and 91.63% on TWOSIDES, highlighting its strong capability in accurately predicting positive samples. It also demonstrates strong performance in statistical significance, with results presented in Supplementary Sections A and B. These findings demonstrate that CMCL-DDI achieves remarkable performance in DDI prediction tasks involving known drugs. As shown in Fig 3, under the warm-start setting, where all drugs have appeared during training, most baseline models achieve relatively higher scores. Yet, CMCL-DDI still maintains a consistent lead across both datasets, reflecting its overall modeling strength regardless of data sparsity.

**Table 3:** The performance of CMCL-DDI and baselines on two datasets in the warm-start setting (%).

Model	DrugBank				Twosides			
	ACC	AUROC	AUPRC	F1	ACC	AUROC	AUPRC	F1
MHCADDI	83.80 ± 0.56	91.16 ± 0.41	89.26 ± 1.63	85.06 ± 1.35	-	88.20 ± 1.32	-	-
SSI-DDI	96.33 ± 0.81	98.95 ± 1.16	98.57 ± 1.39	96.38 ± 0.65	78.20 ± 1.21	85.85 ± 0.88	82.71 ± 1.05	79.81 ± 1.29
MR-GNN	93.23 ± 0.93	97.31 ± 0.81	96.45 ± 0.65	93.39 ± 1.51	85.39 ± 1.29	91.93 ± 1.48	89.32 ± 0.98	86.46 ± 1.62
GMPNN-CS	95.31 ± 1.15	98.45 ± 0.72	97.91 ± 0.83	95.40 ± 1.15	86.96 ± 1.29	92.94 ± 1.03	90.38 ± 0.71	87.85 ± 1.13
GAT-DDI	92.03 ± 0.65	96.28 ± 1.39	94.64 ± 0.88	92.29 ± 1.53	67.32 ± 1.33	75.16 ± 0.95	72.50 ± 1.11	63.70 ± 1.27
DGNN-DDI	96.09 ± 0.72	98.94 ± 1.03	98.51 ± 0.93	96.16 ± 1.12	85.29 ± 1.05	91.92 ± 0.81	89.41 ± 1.09	86.12 ± 0.99
CMCL-DDI(ours)	**98.26 ± 0.38**	**99.23 ± 0.43**	**99.25 ± 0.32**	**97.69 ± 0.39**	**90.25 ± 0.41**	**94.27 ± 0.35**	**91.63 ± 0.53**	**88.93 ± 0.33**

In the cold-start scenario, no drugs are shared between the training and testing sets, meaning that the data are partitioned based on drug identity. This setting evaluates the models’ ability to predict DDIs involving previously unseen drugs. As the models have no prior structural information about the drugs in the testing set, DDI prediction becomes more challenging and demands stronger generalization capabilities [25,44]. Formally, let *G* denote the set of all drugs, *G*_*new*_ represent the set of new drugs and *G*_*old*_ denote the set of drugs used for training. Evidently, $G_{new}\cup G_{old}=G$ and $G_{new}\cap G_{old}=\emptyset$. Finally, we repeated three times and reported the average performance. In each run, we randomly sampled 20% drugs as new drugs to construct different testing sets across 5 runs. Notably, negative samples are generated separately within the training and testing sets based on their respective contained drugs, ensuring consistency with the cold-start setting. Both drug selection and negative sample generation are performed prior to training, guaranteeing that all models are trained, validated, and tested on identical datasets.

**Fig 4:**
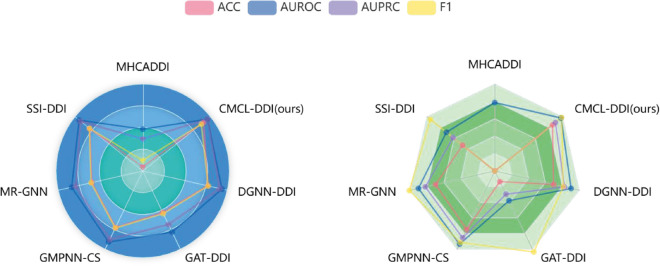
Performance comparison of different models under cold-start setting. The left figure displays results on the DrugBank dataset, and the right figure shows results on the Twosides dataset.

Table 4 presents the average performance of all models over three runs. The cold-start setting markedly reduces the performance of all models; however, CMCL-DDI consistently demonstrates superior results compared to the baselines. Specifically, CMCL-DDI achieves improvements of 1.34% in AUROC on DrugBank and 3.51% on Twosides compared with previous state-of-the-art model, along with F1 score increases of 6.17% and 8.60%, respectively. It also demonstrates strong performance in statistical significance, with results presented in Supplementary Sections C and D. These findings confirm that CMCL-DDI enhances the prediction of DDIs involving previously unseen drugs. Despite the synthetic generation of negative samples, CMCL-DDI maintains strong predictive performance for original positive instances. As illustrated in Fig 4, under the cold-start setting, the performance of all methods drops significantly due to the challenge of predicting interactions involving unseen drugs. Nevertheless, CMCL-DDI shows clear advantages, demonstrating its robustness and generalization capability in such a difficult scenario. In summary, CMCL-DDI achieves state-of-the-art results in both warm-start and cold-start settings.

**Table 4:** The performance of CMCL-DDI and baselines on two datasets in the cold-start setting (%).

Model	DrugBank				Twosides			
	ACC	AUROC	AUPRC	F1	ACC	AUROC	AUPRC	F1
MHCADDI	70.58 ± 1.29	77.84 ± 1.15	76.16 ± 1.09	72.74 ± 0.95	66.50 ± 1.28	72.53 ± 1.16	71.06 ± 0.98	67.21 ± 0.99
SSI-DDI	76.38 ± 1.36	84.23 ± 1.41	84.94 ± 0.92	73.54 ± 1.27	65.40 ± 1.16	73.43 ± 1.35	75.03 ± 1.02	54.12 ± 0.95
MR-GNN	75.99 ± 1.33	84.85 ± 1.29	84.89 ± 0.99	72.30 ± 1.17	67.33 ± 0.81	76.52 ± 1.49	75.25 ± 1.47	59.71 ± 1.12
GMPNN-CS	79.95 ± 1.31	89.34 ± 1.26	89.25 ± 1.15	77.22 ± 1.06	71.57 ± 1.28	81.90 ± 0.93	82.90 ± 1.21	63.83 ± 0.97
GAT-DDI	77.94 ± 1.59	86.58 ± 1.62	85.81 ± 1.42	75.28 ± 0.93	71.55 ± 0.96	80.71 ± 1.35	80.44 ± 1.09	65.91 ± 1.53
DGNN-DDI	77.07 ± 1.41	86.35 ± 1.06	86.97 ± 0.97	73.03 ± 1.12	70.31 ± 1.17	85.12 ± 1.02	83.71 ± 1.38	59.41 ± 1.43
CMCL-DDI(ours)	**88.15 ± 0.31**	**87.69 ± 0.26**	**87.31 ± 0.45**	**83.39 ± 0.39**	**80.21 ± 0.42**	**88.63 ± 0.23**	**85.92 ± 0.35**	**75.81 ± 0.21**

### Ablation study

To further investigate the contribution of each module in CMCL-DDI, we perform an ablation study under the cold-start setting on DrugBank dataset, which more effectively differentiates the performance of the models. The variants considered in this study are as follows:

- w/o graph view module: This variant removes the graph-based molecular representation and relies solely on the sequence view for encoding drug information, aiming to assess the role of structural information in DDI prediction.
- w/o sequence view module: This variant removes the sequence-based molecular representation and utilizes only the graph view to encode drug information, aiming to evaluate the contribution of sequence-level features to the overall performance.

The results of the ablation study in Table 5 show that each module contributes significantly to the overall performance of CMCL-DDI. When the graph view module is removed (w/o graph view module), there is a marked decline in both AUROC and F1 scores, with AUROC dropping by 5.74% and F1 by 4.72%. This indicates that the graph-based molecular representation, which captures structural information, is crucial for accurately modeling the interactions between drugs. On the other hand, removing the sequence view module (w/o sequence view module) also leads to a significant performance reduction, with AUROC and F1 decreasing by 7.82% and 7.27%, respectively. This suggests that sequence-level features, which capture sequential patterns in the drug structure, provide complementary information that enhances the predictive accuracy.

**Table 5:** Ablation study performance of CMCL-DDI.

Model	ACC	AUROC	AUPRC	F1
CMCL-DDI(w/o graph view module)	82.92	81.95	80.95	78.67
CMCL-DDI(w/o sequence view module)	80.33	80.25	80.18	76.12
CMCL-DDI	**88.15**	**87.69**	**87.31**	**83.39**

### Parameter sensitivity studies

In this study, we systematically investigate the impact of various hyperparameters on the performance of the proposed CMCL-DDI model, including the dimension of feature embeddings and the number of attention heads. We conduct parameter sensitivity studies on the DrugBank dataset in a cold-start setting.

#### The dimension of feature embeddings.

**Fig 5:**
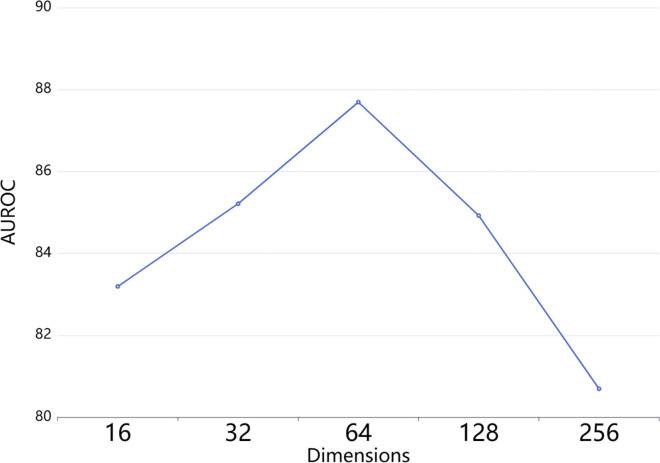
Sensitivity analysis on the dimension of feature embeddings.

We evaluate the impact of the dimension of feature embeddings by experimenting with five different settings: 16, 32, 64, 128, and 256. The results in Fig 5 show that the model achieves the best performance when the embedding dimension is set to 64. Specifically, smaller dimensions such as 16 and 32 lead to insufficient capacity for capturing complex drug representations, resulting in suboptimal performance. In contrast, while larger dimensions like 128 and 256 offer greater representational power, they tend to introduce redundancy and increase the risk of overfitting, ultimately degrading the model’s generalization ability. These findings suggest that setting the feature embedding dimension to 64 strikes a good balance between expressive capacity and generalization, thereby yielding the most favorable results for DDI prediction.

#### The number of attention heads.

**Fig 6:**
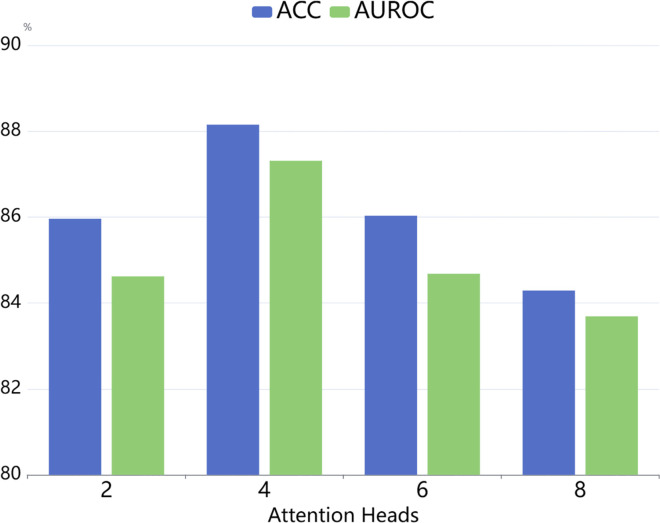
Sensitivity analysis of the number of attention heads.

We further analyze the impact of the number of attention heads on the performance of the CMCL-DDI model. Specifically, we experiment with 2, 4, 6, and 8 heads. As shown in Fig 6, setting the number of attention heads to 4 yields the best performance, achieving an ACC of 88.15% and an AUPRC of 87.31%. Using only 2 heads leads to suboptimal performance due to insufficient capacity to model diverse drug interactions. Conversely, increasing the number of heads to 6 or 8 results in a slight decline in performance, which may be caused by overfitting or the added complexity affecting training stability. These results indicate that using 4 attention heads provides a good balance between representational power and generalization ability in our setting.

### Case study

**Fig 7:**
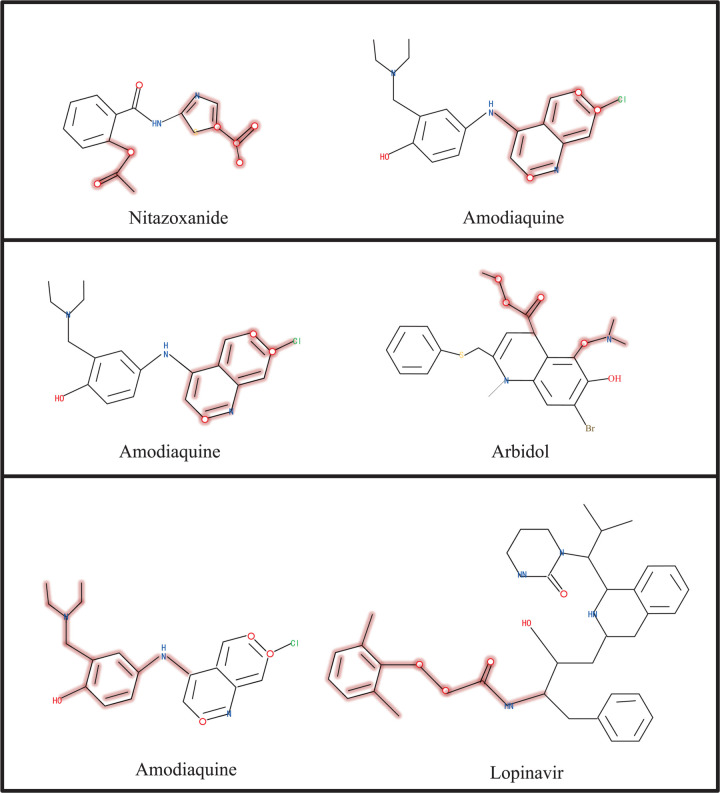
Case studies demonstrating pharmacophores identified by CMCL-DDI in clinically confirmed DDIs.

To assess the effectiveness and interpretability of our proposed model, we examined three drug pairs known to cause drug–drug interactions (DDIs) in Fig 7: Nitazoxanide–Amodiaquine, Amodiaquine–Arbidol, and Amodiaquine–Lopinavir. These combinations have been reported to induce DDIs through mechanisms such as enzyme inhibition or metabolic interference. Our model successfully identified pharmacologically relevant substructures in these pairs that are associated with interaction mechanisms. For example, in the Nitazoxanide–Amodiaquine pair, the model captured structural features related to metabolic inhibition. In the Amodiaquine–Arbidol and Amodiaquine–Lopinavir pairs, it effectively focused on interaction-prone regions. These findings demonstrate the model’s ability not only to accurately predict DDIs but also to provide chemically meaningful insights, which is essential for enhancing the reliability and transparency of DDI prediction in drug development.

## Discussion

The experimental results clearly demonstrate the effectiveness of CMCL-DDI in drug-drug interaction (DDI) prediction tasks across multiple benchmark datasets. The superior performance of our model compared to existing baselines highlights the value of jointly modeling molecular structural and sequential semantics through a cross-view contrastive learning strategy.

One key factor contributing to this improvement is the pharmacophore-aware graph encoder, which allows CMCL-DDI to incorporate functional substructures known to influence drug activity. Unlike prior GNN-based models that process atomic-level graphs indiscriminately, our approach selectively aggregates subgraph features guided by pharmacophore annotations, leading to more biologically meaningful representations. This structural inductive bias not only enhances the interpretability of the model but also improves its robustness when facing structurally diverse compounds.

Additionally, our use of SMILES sequence encoders preserves complementary linear chemical information, which is often neglected in graph-only approaches. The cross-view mutual contrastive learning mechanism ensures that both structural and sequential modalities align in a shared latent space. This alignment facilitates mutual representation refinement, enabling the model to benefit from the unique strengths of each modality. In particular, the contrastive objective promotes the learning of view-invariant features, enhancing generalization on unseen drug pairs.

Furthermore, the cross-attention fusion module explicitly learns the inter-dependencies between molecular structural and sequential representations, enabling more effective integration of heterogeneous information. This module facilitates bidirectional information exchange between the two feature spaces.

Despite these promising results, several limitations remain. First, while our method focuses on binary DDI classification, real-world scenarios often involve multi-type or context-specific interactions. Extending CMCL-DDI to support multi-relational DDI prediction and incorporating external knowledge sources (e.g., drug-target interactions, side effects) would be valuable directions. Second, interpretability at the clinical decision level remains an open challenge. While our architecture supports structural interpretability via pharmacophore embeddings, future studies could investigate model explanations aligned with clinical pharmacology. Finally, CMCL-DDI treats drug pairs independently, overlooking complex interactions in polypharmacy scenarios. Incorporating multi-drug networks or patient-specific contexts could further enhance clinical relevance.

In summary, CMCL-DDI bridges structural and sequential modalities with contrastive mutual learning and attention-guided fusion, achieving state-of-the-art performance in DDI prediction. This framework offers a promising step toward more interpretable, accurate, and robust computational pharmacology systems.

## Conclusion

In this study, we introduced the Cross-view Contrastive Learning framework for DDI Prediction (CMCL-DDI), which integrates both the structural representation of drug molecular graphs and the sequential information from SMILES strings. By leveraging pharmacophore-based graph encoding, our model captures crucial drug–drug interaction patterns that traditional graph-based models may overlook. The contrastive learning strategy effectively aligns and integrates heterogeneous drug features, leading to more comprehensive DDI discovery. Experimental results show that CMCL-DDI outperforms state-of-the-art baselines, demonstrating its potential for enhancing drug safety and efficacy. However, the model currently lacks consideration of the molecular image-based view, which could offer additional insights into drug interactions. Future work will focus on incorporating molecular image data to further improve DDI prediction performance.

## Supporting information

S1 Table Statistical significance analysis of performance differences among CMCL-DDI and baseline models on the DrugBank dataset under the warm-start setting using the Kruskal-Wallis test. (PDF)

S2 Table Pairwise statistical comparison between CMCL-DDI and baseline models on the DrugBank dataset under the warm-start setting using the Mann-Whitney U test with Holm-Bonferroni correction. (PDF)

S3 Table Statistical significance analysis of performance differences among CMCL-DDI and baseline models on the Twosides dataset under the warm-start setting using the Kruskal-Wallis test. (PDF)

S4 Table Pairwise statistical comparison between CMCL-DDI and baseline models on the Twosides dataset under the warm-start setting using the Mann-Whitney U test with Holm-Bonferroni correction. (PDF)

S5 Table Statistical significance analysis of performance differences among CMCL-DDI and baseline models on the DrugBank dataset under the cold-start setting using the Kruskal-Wallis test. (PDF)

S6 Table Pairwise statistical comparison between CMCL-DDI and baseline models on the DrugBank dataset under the cold-start setting using the Mann-Whitney U test with Holm-Bonferroni correction. (PDF)

S7 Table Statistical significance analysis of performance differences among CMCL-DDI and baseline models on the Twosides dataset under the cold-start setting using the Kruskal-Wallis test. (PDF)

S8 Table Pairwise statistical comparison between CMCL-DDI and baseline models on the Twosides dataset under the cold-start setting using the Mann-Whitney U test with Holm-Bonferroni correction. (PDF)

## References

[1] Zhong Y, Li G, Yang J, Zheng H, Yu Y, Zhang J, et al. Learning motif-based graphs for drug–drug interaction prediction via local–global self-attention. Nat Mach Intell. 2024;6(9):1094–105. doi: 10.1038/s42256-024-00888-6

[2] Takeda T, Hao M, Cheng T, Bryant SH, Wang Y. Predicting drug-drug interactions through drug structural similarities and interaction networks incorporating pharmacokinetics and pharmacodynamics knowledge. J Cheminform. 2017;9:16. doi: 10.1186/s13321-017-0200-8 28316654 PMC5340788

[3] Huang D, Jiang Z, Zou L, Li L. Drug–drug interaction extraction from biomedical literature using support vector machine and long short term memory networks. Information Sciences. 2017;415–416:100–9. doi: 10.1016/j.ins.2017.06.021

[4] Chen X, Ren B, Chen M, Wang Q, Zhang L, Yan G. NLLSS: predicting synergistic drug combinations based on semi-supervised learning. PLoS Comput Biol. 2016;12(7):e1004975. doi: 10.1371/journal.pcbi.1004975 27415801 PMC4945015

[5] Han K, Jeng EE, Hess GT, Morgens DW, Li A, Bassik MC. Synergistic drug combinations for cancer identified in a CRISPR screen for pairwise genetic interactions. Nat Biotechnol. 2017;35(5):463–74. doi: 10.1038/nbt.3834 28319085 PMC5557292

[6] Sun X, Dong K, Ma L, Sutcliffe R, He F, Chen S, et al. Drug-drug interaction extraction via recurrent hybrid convolutional neural networks with an improved focal loss. Entropy (Basel). 2019;21(1):37. doi: 10.3390/e21010037 33266753 PMC7514143

[7] Gulikers JL, Otten L-S, Hendriks LEL, Winckers K, Henskens Y, Leentjens J, et al. Proactive monitoring of drug-drug interactions between direct oral anticoagulants and small-molecule inhibitors in patients with non-small cell lung cancer. Br J Cancer. 2024;131(3):481–90. doi: 10.1038/s41416-024-02744-1 38862741 PMC11300802

[8] Jordan MI, Mitchell TM. Machine learning: trends, perspectives, and prospects. Science. 2015;349(6245):255–60. doi: 10.1126/science.aaa8415 26185243

[9] Wu Z, Shangguan D, Huang Q, Wang Y-K. Drug metabolism and transport mediated the hepatotoxicity of Pleuropterus multiflorus root: a review. Drug Metab Rev. 2024;56(4):349–58. doi: 10.1080/03602532.2024.2405163 39350738

[10] Kipf TN, Welling M. Semi-supervised classification with graph convolutional networks. arXiv preprint 2016. doi: arXiv:160902907

[11] Veličković P, Cucurull G, Casanova A, Romero A, Lio P, Bengio Y. Graph attention networks. arXiv preprint 2017. https://arxiv.org/abs/1710.10903

[12] Xu K, Hu W, Leskovec J, Jegelka S. How powerful are graph neural networks? arXiv preprint 2018. https://arxiv.org/abs/1810.00826

[13] Ryu JY, Kim HU, Lee SY. Deep learning improves prediction of drug-drug and drug-food interactions. Proc Natl Acad Sci U S A. 2018;115(18):E4304–11. doi: 10.1073/pnas.1803294115 29666228 PMC5939113

[14] Sun M, Wang F, Elemento O, Zhou J. Structure-based drug-drug interaction detection via expressive graph convolutional networks and deep sets (student abstract). AAAI. 2020;34(10):13927–8. doi: 10.1609/aaai.v34i10.7236

[15] Hong Y, Luo P, Jin S, Liu X. LaGAT: link-aware graph attention network for drug-drug interaction prediction. Bioinformatics. 2022;38(24):5406–12. doi: 10.1093/bioinformatics/btac682 36271850 PMC9750103

[16] Li Z, Tu X, Chen Y, Lin W. HetDDI: a pre-trained heterogeneous graph neural network model for drug-drug interaction prediction. Brief Bioinform. 2023;24(6):bbad385. doi: 10.1093/bib/bbad385 37903412

[17] Vo TH, Nguyen NTK, Le NQK. Improved prediction of drug-drug interactions using ensemble deep neural networks. Medicine in Drug Discovery. 2023;17:100149. doi: 10.1016/j.medidd.2022.100149

[18] Weininger D. SMILES, a chemical language and information system. 1. Introduction to methodology and encoding rules. J Chem Inf Comput Sci. 1988;28(1):31–6. doi: 10.1021/ci00057a005

[19] Landrum G. RDKit: a software suite for cheminformatics, computational chemistry, and predictive modeling. J Cheminform. 2013;8(31.10):5281.

[20] Wang Y, Min Y, Chen X, Wu J. Multi-view graph contrastive representation learning for drug-drug interaction prediction. In: Proceedings of the Web Conference 2021 . 2021. p. 2921–33. 10.1145/3442381.3449786

[21] Lin K, Kang L, Yang F, Lu P, Lu J. MFDA: Multiview fusion based on dual-level attention for drug interaction prediction. Front Pharmacol. 2022;13:1021329. doi: 10.3389/fphar.2022.1021329 36278200 PMC9584567

[22] Zhang R, Wang X, Wang P, Meng Z, Cui W, Zhou Y. HTCL-DDI: a hierarchical triple-view contrastive learning framework for drug-drug interaction prediction. Brief Bioinform. 2023;24(6):bbad324. doi: 10.1093/bib/bbad324 37742052

[23] Gan Y, Liu W, Xu G, Yan C, Zou G. DMFDDI: deep multimodal fusion for drug-drug interaction prediction. Brief Bioinform. 2023;24(6):bbad397. doi: 10.1093/bib/bbad397 37930025

[24] Deac A, Huang YH, Veličković P, Liò P, Tang J. Drug-drug adverse effect prediction with graph co-attention. arXiv preprint 2019. https://arxiv.org/abs/1905.00534

[25] Wang J, Liu X, Shen S, Deng L, Liu H. DeepDDS: deep graph neural network with attention mechanism to predict synergistic drug combinations. Brief Bioinform. 2022;23(1):bbab390. doi: 10.1093/bib/bbab390 34571537

[26] Zhang X, Wang G, Meng X, Wang S, Zhang Y, Rodriguez-Paton A, et al. Molormer: a lightweight self-attention-based method focused on spatial structure of molecular graph for drug-drug interactions prediction. Brief Bioinform. 2022;23(5):bbac296. doi: 10.1093/bib/bbac296 35849817

[27] Wang H, Lian D, Zhang Y, Qin L, Lin X. Gognn: graph of graphs neural network for predicting structured entity interactions. arXiv preprint 2020. https://arxiv.org/abs/2005.05537

[28] Nyamabo AK, Yu H, Shi J-Y. SSI-DDI: substructure-substructure interactions for drug-drug interaction prediction. Brief Bioinform. 2021;22(6):bbab133. doi: 10.1093/bib/bbab133 33951725

[29] Yang Z, Zhong W, Lv Q, Yu-Chian Chen C. Learning size-adaptive molecular substructures for explainable drug-drug interaction prediction by substructure-aware graph neural network. Chem Sci. 2022;13(29):8693–703. doi: 10.1039/d2sc02023h 35974769 PMC9337739

[30] Li Z, Zhu S, Shao B, Zeng X, Wang T, Liu T-Y. DSN-DDI: an accurate and generalized framework for drug-drug interaction prediction by dual-view representation learning. Brief Bioinform. 2023;24(1):bbac597. doi: 10.1093/bib/bbac597 36592061

[31] Nyamabo AK, Yu H, Shi J-Y. SSI-DDI: substructure-substructure interactions for drug-drug interaction prediction. Brief Bioinform. 2021;22(6):bbab133. doi: 10.1093/bib/bbab133 33951725

[32] Yu H, Zhao S, Shi J. STNN-DDI: a substructure-aware tensor neural network to predict drug-drug interactions. Brief Bioinform. 2022;23(4):bbac209. doi: 10.1093/bib/bbac209 35667078

[33] He C, Liu Y, Li H, Zhang H, Mao Y, Qin X, et al. Multi-type feature fusion based on graph neural network for drug-drug interaction prediction. BMC Bioinformatics. 2022;23(1):224. doi: 10.1186/s12859-022-04763-2 35689200 PMC9188183

[34] Pang S, Zhang Y, Song T, Zhang X, Wang X, Rodriguez-Patón A. AMDE: a novel attention-mechanism-based multidimensional feature encoder for drug-drug interaction prediction. Brief Bioinform. 2022;23(1):bbab545. doi: 10.1093/bib/bbab545 34965586

[35] He H, Chen G, Yu-Chian Chen C. 3DGT-DDI: 3D graph and text based neural network for drug-drug interaction prediction. Brief Bioinform. 2022;23(3):bbac134. doi: 10.1093/bib/bbac134 35511112

[36] Schütt K, Kindermans PJ, Sauceda Felix HE, Chmiela S, Tkatchenko A, Müller KR. Schnet: a continuous-filter convolutional neural network for modeling quantum interactions. Advances in Neural Information Processing Systems. 2017;30.

[37] Wang Y, Min Y, Chen X, Wu J. Multi-view graph contrastive representation learning for drug-drug interaction prediction. In: Proceedings of the Web Conference 2021 . 2021. p. 2921–33. 10.1145/3442381.3449786

[38] Nowozin S, Cseke B, Tomioka R. f-gan: Training generative neural samplers using variational divergence minimization. Advances in Neural Information Processing Systems. 2016;29.

[39] Li Z, Zhu S, Shao B, Zeng X, Wang T, Liu T-Y. DSN-DDI: an accurate and generalized framework for drug-drug interaction prediction by dual-view representation learning. Brief Bioinform. 2023;24(1):bbac597. doi: 10.1093/bib/bbac597 36592061

[40] Degen J, Wegscheid-Gerlach C, Zaliani A, Rarey M. On the art of compiling and using “drug-like” chemical fragment spaces. ChemMedChem. 2008;3(10):1503–7. doi: 10.1002/cmdc.200800178 18792903

[41] Li H, Wu X-J. CrossFuse: a novel cross attention mechanism based infrared and visible image fusion approach. Information Fusion. 2024;103:102147. doi: 10.1016/j.inffus.2023.102147

[42] Zheng J, Liu H, Feng Y, Xu J, Zhao L. CASF-Net: Cross-attention and cross-scale fusion network for medical image segmentation. Comput Methods Programs Biomed. 2023;229:107307. doi: 10.1016/j.cmpb.2022.107307 36571889

[43] Wang J, Yu L, Tian S. Cross-attention interaction learning network for multi-model image fusion via transformer. Engineering Applications of Artificial Intelligence. 2025;139:109583. doi: 10.1016/j.engappai.2024.109583

[44] Wishart DS, Feunang YD, Guo AC, Lo EJ, Marcu A, Grant JR, et al. DrugBank 5.0: a major update to the DrugBank database for 2018 . Nucleic Acids Res. 2018;46(D1):D1074–82. doi: 10.1093/nar/gkx1037 29126136 PMC5753335

[45] Zitnik M, Agrawal M, Leskovec J. Modeling polypharmacy side effects with graph convolutional networks. Bioinformatics. 2018;34(13):i457–66. doi: 10.1093/bioinformatics/bty294 29949996 PMC6022705

[46] Deac A, Huang YH, Veličković P, Liò P, Tang J. Drug-drug adverse effect prediction with graph co-attention. arXiv preprint 2019. https://arxiv.org/abs/1905.00534

[47] Nyamabo AK, Yu H, Shi J-Y. SSI-DDI: substructure-substructure interactions for drug-drug interaction prediction. Brief Bioinform. 2021;22(6):bbab133. doi: 10.1093/bib/bbab133 33951725

[48] Xu N, Wang P, Chen L, Tao J, Zhao J. Mr-gnn: multi-resolution and dual graph neural network for predicting structured entity interactions. arXiv preprint 2019. https://arxiv.org/abs/1905.09558

[49] Nyamabo AK, Yu H, Liu Z, Shi J-Y. Drug-drug interaction prediction with learnable size-adaptive molecular substructures. Brief Bioinform. 2022;23(1):bbab441. doi: 10.1093/bib/bbab441 34695842

[50] Veličković P, Cucurull G, Casanova A, Romero A, Lio P, Bengio Y. Graph attention networks. arXiv preprint 2017. https://arxiv.org/abs/1710.10903

[51] Ma M, Lei X. A dual graph neural network for drug-drug interactions prediction based on molecular structure and interactions. PLoS Comput Biol. 2023;19(1):e1010812. doi: 10.1371/journal.pcbi.1010812 36701288 PMC9879511

