# Supplementary material for: CMCL-DDI: Pharmacophore-aware cross-view contrastive learning for drug-drug interaction prediction
Source: PLoS One. 2026 Feb 23;21(2):e0341952. doi: 10.1371/journal.pone.0341952 (PMC12928573; doi:10.1371/journal.pone.0341952)
Supplement: S1 Table — (PDF) [file pone.0341952.s001.pdf]

S1 Table. Statistical significance analysis of performance differences among CMCL-DDI and baseline models on the DrugBank dataset under the warm-start setting using the Kruskal–Wallis test.

Metric	H statistic	p-value
ACC	21.47	0.0003
AUROC	18.92	0.0011
AUPRC	20.35	0.0005
F1	19.77	0.0007
