# Supplementary material for: CMCL-DDI: Pharmacophore-aware cross-view contrastive learning for drug-drug interaction prediction
Source: PLoS One. 2026 Feb 23;21(2):e0341952. doi: 10.1371/journal.pone.0341952 (PMC12928573; doi:10.1371/journal.pone.0341952)
Supplement: S3 Table — (PDF) [file pone.0341952.s003.pdf]

S3 Table. Statistical significance analysis of performance differences among CMCL-DDI and baseline models on the Twosides dataset under the warm-start setting using the Kruskal-Wallis test.

**Metric**	**H statistic**	**p-value**
ACC	17.86	0.0025
AUROC	19.34	0.0016
AUPRC	18.97	0.0019
F1	20.12	0.0011
