# Supplementary material for: CMCL-DDI: Pharmacophore-aware cross-view contrastive learning for drug-drug interaction prediction
Source: PLoS One. 2026 Feb 23;21(2):e0341952. doi: 10.1371/journal.pone.0341952 (PMC12928573; doi:10.1371/journal.pone.0341952)
Supplement: S4 Table — (PDF) [file pone.0341952.s004.pdf]

S4 Table. Pairwise statistical comparison between CMCL-DDI and baseline models on the Twosides dataset under the warm-start setting using the Mann-Whitney U test with Holm-Bonferroni correction.

Metric	Comparison	U statistic	p-value (raw)	p-value (Holm)	Significance
ACC	CMCL-DDI vs MHCADDI	23.0	0.0042	0.0126	Yes
ACC	CMCL-DDI vs SSI-DDI	24.0	0.0048	0.0144	Yes
ACC	CMCL-DDI vs MR-GNN	22.5	0.0036	0.0108	Yes
ACC	CMCL-DDI vs GMPNN-CS	23.5	0.0040	0.0120	Yes
ACC	CMCL-DDI vs GAT-DDI	21.0	0.0031	0.0093	Yes
ACC	CMCL-DDI vs DGNN-DDI	24.5	0.0046	0.0138	Yes
AUROC	CMCL-DDI vs MHCADDI	22.5	0.0039	0.0117	Yes
AUROC	CMCL-DDI vs SSI-DDI	24.5	0.0045	0.0135	Yes
AUROC	CMCL-DDI vs MR-GNN	22.0	0.0033	0.0099	Yes
AUROC	CMCL-DDI vs GMPNN-CS	23.0	0.0038	0.0114	Yes
AUROC	CMCL-DDI vs GAT-DDI	20.5	0.0029	0.0087	Yes
AUROC	CMCL-DDI vs DGNN-DDI	24.0	0.0043	0.0129	Yes
AUPRC	CMCL-DDI vs MHCADDI	24.5	0.0041	0.0123	Yes
AUPRC	CMCL-DDI vs SSI-DDI	24.0	0.0039	0.0117	Yes
AUPRC	CMCL-DDI vs MR-GNN	22.0	0.0030	0.0090	Yes
AUPRC	CMCL-DDI vs GMPNN-CS	23.0	0.0035	0.0105	Yes
AUPRC	CMCL-DDI vs GAT-DDI	20.0	0.0026	0.0078	Yes
AUPRC	CMCL-DDI vs DGNN-DDI	23.5	0.0037	0.0111	Yes
F1	CMCL-DDI vs MHCADDI	23.5	0.0038	0.0114	Yes
F1	CMCL-DDI vs SSI-DDI	24.0	0.0040	0.0120	Yes
F1	CMCL-DDI vs MR-GNN	22.5	0.0032	0.0096	Yes
F1	CMCL-DDI vs GMPNN-CS	23.5	0.0036	0.0108	Yes
F1	CMCL-DDI vs GAT-DDI	21.0	0.0028	0.0084	Yes
F1	CMCL-DDI vs DGNN-DDI	24.5	0.0042	0.0126	Yes
