# Supplementary material for: CMCL-DDI: Pharmacophore-aware cross-view contrastive learning for drug-drug interaction prediction
Source: PLoS One. 2026 Feb 23;21(2):e0341952. doi: 10.1371/journal.pone.0341952 (PMC12928573; doi:10.1371/journal.pone.0341952)
Supplement: S5 Table — (PDF) [file pone.0341952.s005.pdf]

S5 Table. Statistical significance analysis of performance differences among CMCL-DDI and baseline models on the DrugBank dataset under the cold-start setting using the Kruskal-Wallis test.

Metric	H statistic	p-value
ACC	22.84	0.0002
AUROC	20.17	0.0006
AUPRC	19.63	0.0008
F1	21.05	0.0004
