# Supplementary material for: CMCL-DDI: Pharmacophore-aware cross-view contrastive learning for drug-drug interaction prediction
Source: PLoS One. 2026 Feb 23;21(2):e0341952. doi: 10.1371/journal.pone.0341952 (PMC12928573; doi:10.1371/journal.pone.0341952)
Supplement: S6 Table — (PDF) [file pone.0341952.s006.pdf]

S6 Table. Pairwise statistical comparison between CMCL-DDI and baseline models on the DrugBank dataset under the cold-start setting using the Mann-Whitney U test with Holm-Bonferroni correction.

Metric	Comparison	U statistic	p-value (raw)	p-value (Holm)	Significance
ACC	CMCL-DDI vs MHCADDI	25.0	0.0042	0.0126	Yes
ACC	CMCL-DDI vs SSI-DDI	24.0	0.0050	0.0150	Yes
ACC	CMCL-DDI vs MR-GNN	23.5	0.0058	0.0174	Yes
ACC	CMCL-DDI vs GMPNN-CS	24.0	0.0052	0.0156	Yes
ACC	CMCL-DDI vs GAT-DDI	22.5	0.0065	0.0195	Yes
ACC	CMCL-DDI vs DGNN-DDI	23.0	0.0060	0.0180	Yes
AUROC	CMCL-DDI vs MHCADDI	24.0	0.0050	0.0150	Yes
AUROC	CMCL-DDI vs SSI-DDI	23.5	0.0055	0.0165	Yes
AUROC	CMCL-DDI vs MR-GNN	23.0	0.0060	0.0180	Yes
AUROC	CMCL-DDI vs GMPNN-CS	24.5	0.0048	0.0144	Yes
AUROC	CMCL-DDI vs GAT-DDI	22.0	0.0068	0.0204	Yes
AUROC	CMCL-DDI vs DGNN-DDI	23.5	0.0055	0.0165	Yes
AUPRC	CMCL-DDI vs MHCADDI	25.0	0.0045	0.0135	Yes
AUPRC	CMCL-DDI vs SSI-DDI	24.0	0.0053	0.0159	Yes
AUPRC	CMCL-DDI vs MR-GNN	23.0	0.0062	0.0186	Yes
AUPRC	CMCL-DDI vs GMPNN-CS	23.5	0.0057	0.0171	Yes
AUPRC	CMCL-DDI vs GAT-DDI	21.5	0.0072	0.0216	Yes
AUPRC	CMCL-DDI vs DGNN-DDI	23.0	0.0060	0.0180	Yes
F1	CMCL-DDI vs MHCADDI	24.5	0.0049	0.0147	Yes
F1	CMCL-DDI vs SSI-DDI	23.5	0.0057	0.0171	Yes
F1	CMCL-DDI vs MR-GNN	23.0	0.0062	0.0186	Yes
F1	CMCL-DDI vs GMPNN-CS	24.0	0.0051	0.0153	Yes
F1	CMCL-DDI vs GAT-DDI	22.0	0.0068	0.0204	Yes
F1	CMCL-DDI vs DGNN-DDI	23.5	0.0057	0.0171	Yes
