# Supplementary material for: CMCL-DDI: Pharmacophore-aware cross-view contrastive learning for drug-drug interaction prediction
Source: PLoS One. 2026 Feb 23;21(2):e0341952. doi: 10.1371/journal.pone.0341952 (PMC12928573; doi:10.1371/journal.pone.0341952)
Supplement: S7 Table — (PDF) [file pone.0341952.s007.pdf]

S7 Table. Statistical significance analysis of performance differences among CMCL-DDI and baseline models on the Twosides dataset under the cold-start setting using the Kruskal-Wallis test.

**Metric**	**H statistic**	**p-value**
ACC	22.41	0.0012
AUROC	24.12	0.0009
AUPRC	23.17	0.0010
F1	24.85	0.0007
