# Supplementary material for: CMCL-DDI: Pharmacophore-aware cross-view contrastive learning for drug-drug interaction prediction
Source: PLoS One. 2026 Feb 23;21(2):e0341952. doi: 10.1371/journal.pone.0341952 (PMC12928573; doi:10.1371/journal.pone.0341952)
Supplement: S8 Table — (PDF) [file pone.0341952.s008.pdf]

S8 Table. Pairwise statistical comparison between CMCL-DDI and baseline models on the Twosides dataset under the cold-start setting using the Mann-Whitney U test with Holm-Bonferroni correction.

Metric	Comparison	U statistic	p-value (raw)	p-value (Holm)	Significance
ACC	CMCL-DDI vs MHCADDI	23.5	0.0032	0.0096	Yes
ACC	CMCL-DDI vs SSI-DDI	25.0	0.0041	0.0123	Yes
ACC	CMCL-DDI vs MR-GNN	24.0	0.0045	0.0135	Yes
ACC	CMCL-DDI vs GMPNN-CS	23.5	0.0048	0.0144	Yes
ACC	CMCL-DDI vs GAT-DDI	22.0	0.0053	0.0159	Yes
ACC	CMCL-DDI vs DGNN-DDI	24.5	0.0044	0.0132	Yes
AUROC	CMCL-DDI vs MHCADDI	25.0	0.0040	0.0120	Yes
AUROC	CMCL-DDI vs SSI-DDI	23.0	0.0046	0.0138	Yes
AUROC	CMCL-DDI vs MR-GNN	24.5	0.0042	0.0126	Yes
AUROC	CMCL-DDI vs GMPNN-CS	23.0	0.0049	0.0147	Yes
AUROC	CMCL-DDI vs GAT-DDI	21.5	0.0056	0.0168	Yes
AUROC	CMCL-DDI vs DGNN-DDI	25.0	0.0041	0.0123	Yes
AUPRC	CMCL-DDI vs MHCADDI	25.0	0.0043	0.0129	Yes
AUPRC	CMCL-DDI vs SSI-DDI	24.5	0.0045	0.0135	Yes
AUPRC	CMCL-DDI vs MR-GNN	24.0	0.0047	0.0141	Yes
AUPRC	CMCL-DDI vs GMPNN-CS	23.5	0.0049	0.0147	Yes
AUPRC	CMCL-DDI vs GAT-DDI	22.0	0.0054	0.0162	Yes
AUPRC	CMCL-DDI vs DGNN-DDI	25.0	0.0042	0.0126	Yes
F1	CMCL-DDI vs MHCADDI	25.0	0.0041	0.0123	Yes
F1	CMCL-DDI vs SSI-DDI	24.0	0.0046	0.0138	Yes
F1	CMCL-DDI vs MR-GNN	24.5	0.0043	0.0129	Yes
F1	CMCL-DDI vs GMPNN-CS	23.5	0.0048	0.0144	Yes
F1	CMCL-DDI vs GAT-DDI	21.5	0.0057	0.0171	Yes
F1	CMCL-DDI vs DGNN-DDI	25.0	0.0042	0.0126	Yes
